# Evaluation of community-level interventions to increase early initiation of antenatal care in pregnancy: protocol for the Community REACH study, a cluster randomised controlled trial with integrated process and economic evaluations

**DOI:** 10.1186/s13063-018-2526-6

**Published:** 2018-03-05

**Authors:** Mary Sawtell, Lorna Sweeney, Meg Wiggins, Cathryn Salisbury, Sandra Eldridge, Lauren Greenberg, Rachael Hunter, Inderjeet Kaur, Christine McCourt, Bethan Hatherall, Gail Findlay, Joanne Morris, Sandra Reading, Adrian Renton, Ruth Adekoya, Belinda Green, Belinda Harvey, Sarah Latham, Kanta Patel, Logan Vanlessen, Angela Harden

**Affiliations:** 1 Social Science Research Unit (SSRU), UCL Institute of Education, University College London, 18 Woburn Square, London, WC1H 0NR UK; 2 Institute for Health and Human Development, University House, UH250, Stratford Campus, University of East London, Stratford Campus, London, E12 4LZ UK; 3 Centre for Primary Care and Public Health, Blizard Institute, Barts and The London School of Medicine and Dentistry, Yvonne Carter Building, 58 Turner Street, London, E1 2AB UK; 4 Department of Primary Care and Population Health, University College London, Royal Free Medical School, London, NW3 2PF UK; 5 Barts Health NHS Trust, Royal London Hospital, Whitechapel Road, Whitechapel, London, E1 1BZ UK; 6 School of Health Sciences, City University of London, Northampton Square, London, EC1V 0HB UK; 7 Barts Health NHS Trust, Newham University Hospital, Glen Rd, Plaistow, London, E13 8SL UK; 8 University College London Hospitals NHS Foundation Trust, Elizabeth Garrett Anderson Wing, 25 Grafton Way, London, WC1E 6DB UK; 9 The Princess Alexandra Hospital NHS Trust, Hamstel Road, Harlow, Essex, CM20 1QX UK; 10 Homerton University Hospital NHS Foundation Trust, Homerton Row, London, E9 6SR UK; 11 North Middlesex University Hospital NHS Trust, Sterling Way, London, N18 1QX UK; 12 Whittington Health NHS Trust, Magdala Ave, London, N19 5NF UK; 13 Lay Minister, London, UK

**Keywords:** Access to care, Antenatal care, Cluster randomised controlled trial, Community engagement, Co-production, Maternity

## Abstract

**Background:**

The provision of high-quality maternity services is a priority for reducing inequalities in health outcomes for mothers and infants. Best practice includes women having their initial antenatal appointment within the first trimester of pregnancy in order to provide screening and support for healthy lifestyles, well-being and self-care in pregnancy. Previous research has identified inequalities in access to antenatal care, yet there is little evidence on interventions to improve early initiation of antenatal care. The Community REACH trial will assess the effectiveness and cost-effectiveness of engaging communities in the co-production and delivery of an intervention that addresses this issue.

**Methods/design:**

The study design is a matched cluster randomised controlled trial with integrated process and economic evaluations. The unit of randomisation is electoral ward. The intervention will be delivered in 10 wards; 10 comparator wards will have normal practice. The primary outcome is the proportion of pregnant women attending their antenatal booking appointment by the 12th completed week of pregnancy. This and a number of secondary outcomes will be assessed for cohorts of women (*n* = approximately 1450 per arm) who give birth 2–7 and 8–13 months after intervention delivery completion in the included wards, using routinely collected maternity data. Eight hospitals commissioned to provide maternity services in six NHS trusts in north and east London and Essex have been recruited to the study. These trusts will provide anonymised routine data for randomisation and outcomes analysis. The process evaluation will examine intervention implementation, acceptability, reach and possible causal pathways. The economic evaluation will use a cost-consequences analysis and decision model to evaluate the intervention. Targeted community engagement in the research process was a priority.

**Discussion:**

Community REACH aims to increase early initiation of antenatal care using an intervention that is co-produced and delivered by local communities. This pragmatic cluster randomised controlled trial, with integrated process and economic evaluation, aims to rigorously assess the effectiveness of this public health intervention, which is particularly complex due to the required combination of standardisation with local flexibility. It will also answer questions about scalability and generalisability.

**Trial registration:**

ISRCTN registry: registration number 63066975. Registered on 18 August 2015.

**Electronic supplementary material:**

The online version of this article (10.1186/s13063-018-2526-6) contains supplementary material, which is available to authorized users.

## Background

Inequalities in maternal and infant mortality and morbidity are a challenge for public health policy and service delivery worldwide. In the UK the provision of high-quality maternity services is a priority for reducing national inequalities in health outcomes throughout pregnancy, birth and the subsequent life course of the mother and infant [1]. Antenatal care is the first step in maternity service provision for the pregnant woman. Antenatal care refers to the package of health care services provided throughout pregnancy, from conception to the onset of labour, and includes monitoring the health of the woman and fetus, providing medical and psycho-social support, and health promotion [2]. Under-utilisation of antenatal care is associated with adverse pregnancy outcomes including low birth weight, neonatal mortality and maternal mortality [3, 4]. Current national guidelines recommend that women have a first contact (with a midwife or GP) followed by an antenatal care booking appointment with a maternity service within the first trimester of pregnancy (and ideally by 10 weeks), in order to fully benefit from the available screening, interventions and support [5]. At the first contact the focus is on the provision of antenatal information and screening. The ‘booking appointment’ involves a health check and taking a medical history, information on nutrition and exercise, information on and offers of appropriate screening tests, as well as support for well-being and self-care in pregnancy. The booking appointment is also important for identifying women with social and medical risk factors so that these can be appropriately managed throughout the maternity pathway [6].

The timing of the booking appointment is associated with the quality and availability of health services, and the socio-demographic characteristics of pregnant women [2]. The percentage of women who attend their booking appointment by 12 completed weeks of pregnancy within each National Health Service (NHS) maternity service is an indicator used by the UK Department of Health to monitor local and national inequalities in the provision and uptake of antenatal care [7]. Women from minority ethnic groups are less likely to have their booking appointment by the 12th completed week of pregnancy, in comparison to white women [8, 9]. Late booking is also associated with socio-economic deprivation [10]. This delayed initiation of antenatal care for many pregnant women living in marginalised communities is attributed to lower levels of health literacy within these communities (in relation to knowledge regarding the purpose and importance of antenatal care, and understanding of the health care system), along with reduced autonomy, resources and support to make use of the health care services that are available [11–13].

A systematic review by Oakley et al. [2] of interventions to increase early access to antenatal care for socially disadvantaged groups of women concluded that there was a lack of good-quality evidence on the effectiveness of such interventions, with no randomised controlled trials identified in this area. Interventions that were noted by the reviewers as potentially applicable to the UK setting and worthy of further development and examination included community-based programmes, where lay women are trained to provide information on antenatal care and its availability to women within their communities. A review by Hollowell et al. [13], that focussed on barriers to early initiation of antenatal care by women from minority ethnic communities in the UK, called for the development of interventions that promote the purpose and benefit of early and continued antenatal care in ways that take into account the cultural beliefs and practices of groups at risk of late booking. These authors further emphasised the need for information about antenatal care to be provided in a proactive and accessible format for women who may not be familiar with the UK health care system, or who may not speak English as their first language.

### Community engagement to increase early initiation of antenatal care

Community engagement has been broadly defined as the ‘direct or indirect process of involving communities in decision-making and/or in the planning, design, governance and delivery of services, using methods of consultation, collaboration, and/or community control’ [14, 15]. It is thus an umbrella term for a variety of approaches and methods. In recent years, community engagement has been increasingly recognised as important in national policy and strategy documents for health service delivery, public health promotion and the reduction of health inequalities [16, 17]. Theories of community engagement suggest that involving communities as partners in the planning, design and delivery of interventions for health improvement leads to more appropriate and accessible interventions, and increased sense of ownership of the interventions and health outcomes [18]. A recent review of community engagement approaches to reducing health inequalities found them to be effective in improving health behaviours, health consequences, participant self-efficacy and perceived social support for disadvantaged groups [14]. Community engagement strategies that have targeted disadvantaged pregnant women and new mothers have been found to be most effective when peer delivery or collaborative models are used in interventions [19]. Community development is an example of a collaborative model in which communities and other organisations work together to co-produce locally focussed activities, by building on existing relationships and assets within communities.

Despite the recent policy focus on utilising local strengths, knowledge and resources of communities to co-produce and deliver interventions for health and well-being, there have been few trials of the effectiveness of such interventions [20] and community engagement is not yet routinely embedded in mainstream commissioning and practice. Very few studies have looked at community engagement interventions in relation to antenatal care [19].

This paper presents the study protocol (version 2; 22.1.16) for the first cluster randomised controlled trial of a community-centred intervention that seeks to increase early initiation of antenatal care in communities where women are more likely to experience late initiation of antenatal care. The intervention uses community-engagement approaches, including community development and peer delivery, as part of a locally focussed ‘whole systems’ approach, which will engage and mobilise community assets (e.g. local health care professionals such as midwives, nurses and general practitioners (GPs), faith groups, local businesses) and enhance local people’s capabilities to provide advice and information in relation to health within their own communities.

### Development of the Community REACH study

In 2010, members of the study team received UK National Institute for Health Research (NIHR) Programme Development Grant funding (Grant Reference Number RP-PG-1211-20,015) to carry out exploratory research that would lead to the development of a new intervention to improve early initiation of antenatal care in urban settings with social disadvantage and ethnic diversity. We worked in an East London borough which has the largest proportion of births to mothers who were not born in the UK, at 76.4% [21]. Through epidemiological analysis, socio-demographic and clinical predictors of delayed access to antenatal care in this borough were identified [8]. Women identified as most vulnerable to late access included those: from ethnic minority communities; unable to speak English; born outside of the UK; with more than two children. Qualitative research uncovered several barriers to timely antenatal care attendance that corresponded to those identified in the academic literature outlined above [12]. Barriers identified included: difficulties navigating the referral system, especially if women were not already registered with a GP or had limited or no English; lack of understanding regarding the value and benefits of early antenatal care; lack of agency and sense of entitlement to health care. As part of a public engagement focussed research process [22] a stakeholder workshop was held to plan for intervention development. This brought together maternity service users, maternity service managers, local health care commissioners, representatives of community organisations, and the research team. Workshop participants emphasised that the new intervention ought to work collaboratively with women:; building on women’s networks, empowering women, and harnessing local volunteering.

**Fig. 1:**
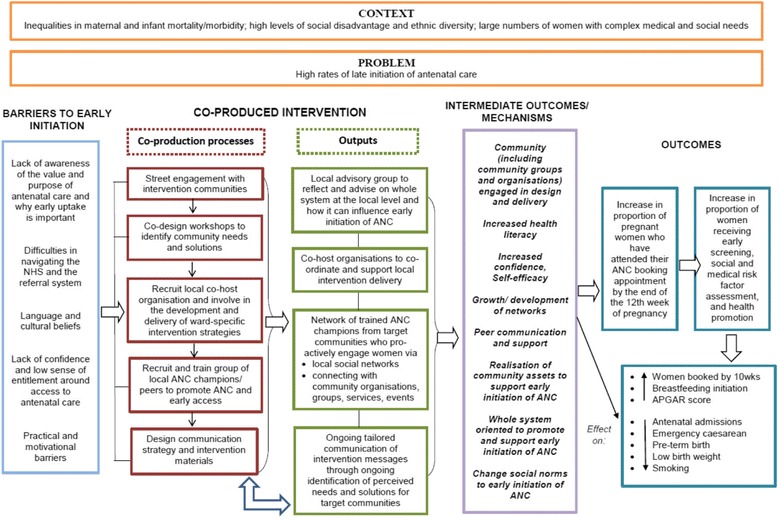
Logic model for the Community REACH intervention

The Community REACH study was developed as a result of this exploratory work with stakeholders. It will test a local, focussed whole systems intervention which aims to (1) raise awareness in local communities of the value of antenatal care and its early uptake and (2) support women in how and when to access care, with the longer term aim to change local social norms which will sustain any increase in women’s early access of antenatal care. The intervention uses a co-production process to engage local communities in: identifying their perceptions/views on the issues and solutions to increase early booking for antenatal care; tailoring the design of the intervention and form and content of key intervention messages; and facilitating the communication of the intervention messages through community self-help and local social networks. Our community engagement team will work with a co-host community organisation already established in each site, to support and implement the intervention at the local level. Peer volunteers, who are women from the local target community, will be recruited and trained for the role of ‘antenatal care champions’ to deliver the intervention messages through engagement with women and wider family members, and local community groups and organisations (ranging from faith groups to pharmacies). A particular focus will be on reaching women from the groups identified through our previous epidemiological analysis to be most vulnerable to late initiation of antenatal care. The theoretical framework for the intervention is informed primarily by the concepts of community engagement and health literacy; the latter is defined as ‘*the cognitive and social skills which determine the motivation and ability of individuals to gain access to, understand and use information in ways which promote and maintain good health*’ [23]. Critically, the concept of health literacy goes beyond the ability to read health information and navigate health services, by increasing access to, and the ability and motivation to act on, health information. Figure 1 displays the theory of change in the form of a logic model for the Community REACH intervention.

The Community REACH study is one component of a wider programme of research, the REACH (Research for Equitable Antenatal Care and Health) Pregnancy Programme. With high priority given to public and practitioner involvement, the Programme is focussed on improving access to, and experience of, antenatal care (Hayes 2012) [22]. The University of East London (UEL) is the lead academic partner. Organisations working with UEL on the Community REACH study include: University College London (UCL) Institute of Education providing trials expertise and management; the Pragmatic Clinical Trials Unit (PCTU) at Queen Mary University of London providing data management, statistical support and quality assurance; UCL conducting the economic evaluation. Uscreates, a design agency with a social focus, supported the initial co-design and communication strategy of the intervention. The study runs from 1 April 2015 (when NHS Research Ethics Committee approval was received) to 11 October 2019 (the end date of the REACH Pregnancy Programme).

This paper was written after funding and approvals were received for the Community REACH study, participating NHS trusts were enrolled, randomisation of study sites and co-design workshops were completed, but prior to the intervention set-up and collection of any trial data.

## Methods/design

### Study design

**Fig. 2:**
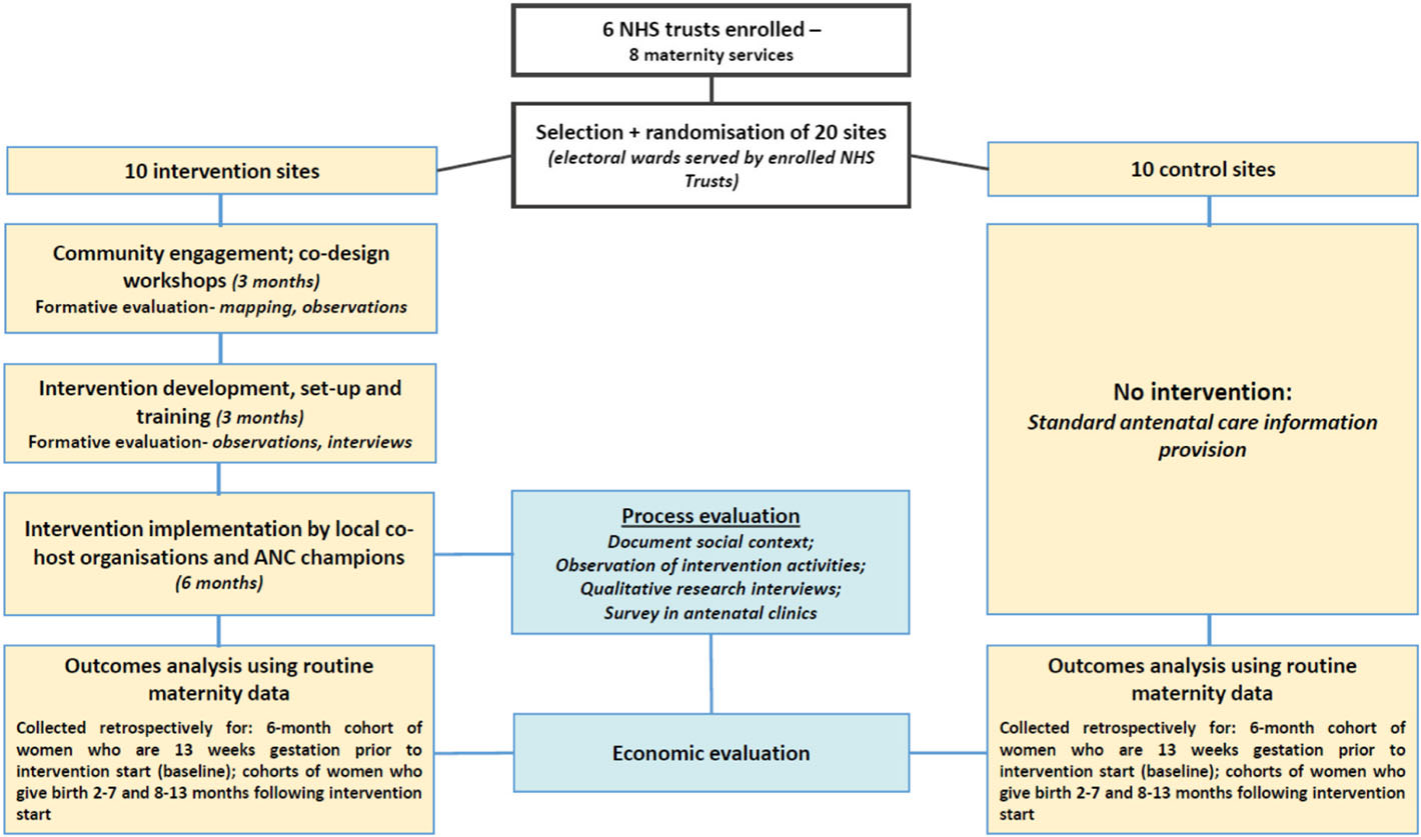
Flow chart of the trial

**Fig. 3:**
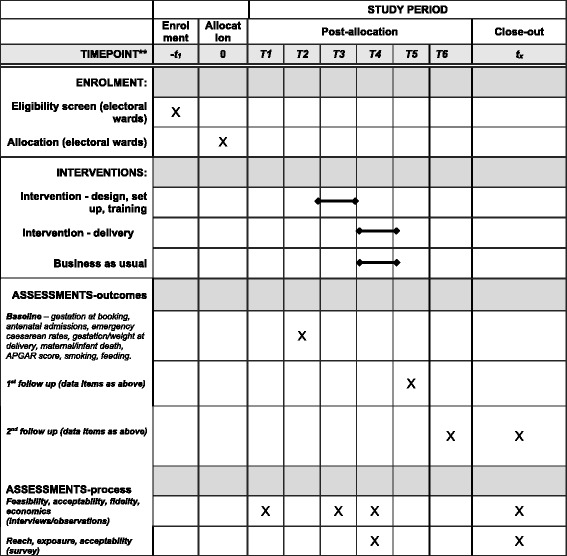
Standard Protocol Items: Recommendations for Interventional Trials (SPIRIT) schedule

The study design is a two-armed, matched cluster randomised controlled trial, with integral process and economic evaluations, see Figs. 2 and 3 and Additional file 1, which present the study flow chart, the Standard Protocol Items: Recommendations for Interventional Trials (SPIRIT) schedule of enrolment, interventions and assessments and SPIRIT Checklist, respectively [24]. This design was selected because the interventions are delivered at the community rather than individual level. The unit (cluster) of randomisation and intervention delivery is electoral ward.^1^ All outcomes are measured using anonymised routinely collected maternity data. The trial includes 20 electoral wards with high delayed rates of initiation of antenatal care, reflecting high rates of inequality in the ward populations. Ten wards were randomised to intervention and 10 to control, matched by these initiation rates and by pattern of use of hospitals in each ward.

Randomised trial design has traditionally required standardisation (or fidelity) of intervention delivery across experimental sites, but the effectiveness of community-based interventions is likely to depend upon whether they are responsive to local needs and contexts [25]. The Community REACH trial is one of a small, but growing, number of cluster-randomised controlled trials of community-led complex public health interventions, where the function and general approach of the interventions are standardised across study sites, but the exact nature of each intervention is adapted at the community level to suit the local context [20].

### Study population

The study population is women in the selected electoral wards who give birth, at a hospital enrolled in the study, over a 12-month period. The timing of this period will be determined by the delivery of the intervention; all women in control and intervention sites will be included if they give birth 2–13 months after the intervention delivery has ended in intervention sites. There will be no active recruitment of participants to this trial as outcomes will be measured using anonymised routinely collected maternity services data. Individuals will be recruited to the integral process evaluation and this is described below.

### Inclusion and exclusion criteria

The unit of randomisation is electoral ward. Inclusion criteria for electoral wards (clusters) are:

- Where the proportion of women who have their first appointment for antenatal care with the maternity service by the 12th completed week of pregnancy is below the NHS national target of 90%, using data collected prior to randomisation over a 6-month period within each hospital
- Where historically the majority of pregnant women have chosen to access the maternity services of the commissioned maternity care provider for the area in which they live (i.e. the service participating in the study), as opposed to services provided by another NHS trust

Exclusion criteria are:

- Where the proportion of women who have their first appointment for antenatal care with the maternity service by the 12th completed week of pregnancy is 90% or above
- Where it is common for pregnant women to access maternity services other than those provided by the hospitals participating in the study

### Recruitment and selection of clusters for randomisation

For both substantive and pragmatic reasons NHS trusts in north and east London and Essex were targeted for recruitment to the study. The inclusion of an out of London area (Essex) is intended to enhance the generalisability of findings. Six NHS trusts agreed to take part, from which eight hospitals providing NHS maternity care (three providers from one large trust) enrolled in the study. NHS trusts were required to commit to providing routine maternity data for randomisation and assessment of outcomes as part of the participation agreement. Any trust unable to commit to this was not able to participate.

The research team identified 20 electoral wards eligible to include in the study, distributed across the geographical areas served by the participating hospitals. This identification process required informatics staff, already responsible for managing routine maternity data in each participating hospital, to extract retrospective ‘gestation at booking’ data for women using the maternity services of these hospitals during a defined recent 6-month period. Routinely this maternity data is organised by postcode. In order to render it non-identifiable for study purposes, the informatics staff converted the postcode data to its corresponding electoral ward name using a postcode-to-ward database sourced from local authorities and provided to the informatics staff by the research team. Informatics staff transferred the data to the research team in a dedicated study database sent in an encrypted form via email. A pilot of this data transfer procedure was completed in one hospital, in order to ensure that all processes could be operationalised and audited.

From the databases of electoral wards, we selected 10 pairs of wards that met the eligibility criteria, ensuring that no wards neighboured one another to minimise intervention/control site contamination. Each pair of wards was matched on the hospitals accessed and baseline rate of antenatal booking by the 12th completed week of pregnancy, categorised as very low (≤ 70%) or low (71–89%). A matched pair consisted of either two ‘very low’ wards or two ‘low’ wards, with similar patterns of hospital usage by women seeking antenatal care. An independent member of the research team, who was not involved in the recruitment of the trusts, oversaw the decision on the final list of 20 sites to ensure that the risk of selection bias was minimised. Each selected site was allocated a unique ID code, which was used in place of electoral ward names for randomisation purposes.

### Randomisation, blinding and retention

Matched randomisation, of the 10 pairs of electoral wards with 1:1 ratio was undertaken remotely by the Pragmatic Clinical Trials Unit (PCTU) at Queen Mary’s University London (QMUL) using Stata software (version 12; StataCorp, College Station, TX, USA). The research team were informed of the results of the allocation by secure email using a password-protected file.

As with most social intervention trials, those involved in delivering the intervention in the clusters (i.e. the electoral wards) cannot be ‘blinded’ to allocation status. Informatics staff in the participating services were given the names of the selected wards in their area but were not actively informed of the results of the randomisation.

Given the nature of this trial, sites cannot drop out of the trial, unless an NHS trust withdraws from the study. We aim to work closely with local site principal investigators (PI) to ensure that this does not occur. The use of routine data for assessment of outcomes means limited missing data can be expected. Data sets provided for the site selection process (described above) confirmed this to be the case; as such no consideration of how to deal with missing data is indicated.

### The intervention

The Community REACH intervention will be delivered in the 10 intervention sites (electoral wards), with each component of the intervention tailored to the local community thereby tapping into local assets and addressing local cultural beliefs and motivational barriers, and community perceived needs and solutions.

#### Phase 1: Mapping, community engagement and co-design

In developing the intervention plans the research team prepared a profile/map for each intervention site, in relation to current referral pathways to antenatal care, demographic information and community assets. Midwives working in the area provided local knowledge on barriers to accessing services. Staff from the design agency Uscreates and members of the UEL community engagement team then spent 2–3 days engaging with local people in each intervention site (through speaking to people at community facilities, marketplaces and other areas of local footfall). Local women and other family members were asked about experiences of antenatal care, perceived importance of antenatal care and their thoughts and opinions on the local area. A co-design workshop was held in each intervention site, facilitated by Uscreates. Local women who had registered their interest in the project during the street engagement, outlined above, were invited to attend, along with representatives from local community organisations and midwives working locally. Attendees participated in exercises to stimulate creativity and worked collaboratively on developing ideas for key messages, materials and events to improve early uptake of antenatal care in the local area.

Workshop participants highlighted the need for greater information about referrals to antenatal care, the services that are available, and the purpose and benefits of antenatal care. They felt that this information ought to be provided through local connections, networks and languages, i.e. women from the community engaging with other local women about antenatal care. In response to workshop outputs, a co-produced community-based intervention will be prepared and implemented in each site, as outlined below:

#### Phase 2: Set-up and training (3 months)

1. The intervention will be centrally co-ordinated by a community engagement team at UEL. A co-host community organisation will be recruited within each intervention site to support the local delivery of the intervention. Co-host organisations must meet certain criteria in order to be involved, for example: experience of managing, supporting and developing outreach teams; demonstrable experience of working with vulnerable groups including black and ethnic minority communitie; strong links to local community groups and organisations and statutory health services
2. Six to eight local people will be recruited in each site as voluntary antenatal care champions, to engage directly and indirectly with women and families, and specific groups and organisations from their community, to raise awareness of the value and benefits of early antenatal care and how and when to access care. The antenatal care champions will receive training for this role (which will involve input from a midwife) on the antenatal care system, including referral pathways and the purpose and benefits of antenatal care. Champions will also be trained in presentation and communication skills, ongoing practice reflection, adult and child safe-guarding, and health and well-being coaching
3. Co-host organisations and antenatal care champions in each site will work collaboratively with the UEL community engagement team and the research team to build on the detailed profiles and mapping of community assets for each intervention site, and to further develop their local outreach plans for intervention implementation, which will engage the whole system within each ward (e.g. schools, children’s centres, GPs, pharmacies, faith groups)
4. A communications strategy and materials for the intervention messages, based on the outputs from the co-design workshops will be developed. The co-host organisations and antenatal care champions will also be part of the decision-making about whether and how the intervention messages ought to be tailored for each site

#### Phase 3: Implementation (6 months)

Implementation of the intervention will take place, in each of the sites, immediately after the development phase. As this will be co-produced and locally tailored, the outreach and engagement plans in each site may vary.

Antenatal care champions are likely to engage with their local communities about antenatal care, particularly women who are vulnerable to later service access, through: presenting and discussing information with groups (e.g. at community events, evening classes, faith groups); one-to-one sessions, where antenatal care champions will engage with local people directly and indirectly in places of high footfall (e.g. GP surgeries, pharmacies, shopping centres); informal, opportunistic outreach, building on existing networks and relationships within the community.

We will use a staggered approach, with implementation taking place first in three ‘pathfinder’ sites before starting in the remaining seven sites, in order that intervention development and delivery can benefit from some initial learning.

### The comparator

Normal maternity care promotion and practice will continue in the 10 electoral wards randomised to the control arm. All participating providers of maternity services are free to engage in additional actions to enhance early booking of antenatal care that affect populations in the electoral wards in either arm of the study.

### Outcome measures

Based on the hypothesis that the intervention will promote earlier booking of care, particularly by those most at risk of late booking (as per the pathways shown in Fig. 1), the primary outcome is the proportion of pregnant women in each ward who have attended their antenatal booking appointment by the end of the 12th completed week of their pregnancy. Secondary outcome measures are: the proportion of women who have attended their antenatal booking appointment by 10 weeks and 0 days of pregnancy; antenatal admissions; emergency caesarean rates; gestation and weight at delivery; smoking at booking appointment and at birth; feeding method at discharge. Outcomes will be measured using anonymised routine hospital data. Outcomes data, covering periods of 6 months, will be extracted by trust informatics staff at three time points. These time points are as follows: time point 1 (baseline) – women giving birth who were at least 13 weeks pregnant at the point that any intervention-related activities commenced; time point 2 (first follow-up) – women giving birth 2–7 months after the end of intervention delivery; time point 3 (second follow-up) – women giving birth 8–13 months after the end of intervention delivery. Further data on age, ethnicity, housing tenure, parity and deprivation will be provided along with the outcomes data.

The study will not require names, NHS numbers, dates of birth, addresses or postcodes. In order to ensure complete anonymisation, trust informatics staff will re-code potentially identifying individual level data; for example, age and ethnicity, into broad categories, ensuring this categorisation does not compromise any proposed analyses. The name of each electoral ward will be replaced by a unique ward-specific ID code that is not known to staff in the PCTU who will be analysing the data. In addition, an unblinded member of the PCTU data management team will liaise with informatics staff in each trust to ensure that secure data transfer methods are understood and used when transferring data from the trust to the PCTU. They will then make the data accessible to their data management colleagues who will remain blind to study site. The data management team will ensure the secure storage of study datasets for statisticians to conduct their analysis. A pilot of the outcomes data transfer process will be conducted across the study sites to ensure that procedures are deliverable by informatics staff and fit for purpose. A collaborative approach involving the research team, informatics staff and site PIs, to finalising the exact list of outcomes and the data transfer processes will be taken.

### Power and sample size

In our Programme Development Grant research, we analysed routine data from the maternity service at Newham University Hospital, which contained the corresponding postcode for each pregnancy in Newham from the period April 2007 to January 2011. We calculated by ward the variation in cluster size and the proportion accessing antenatal care by the 12th completed week of pregnancy as the basis for estimation of intra-cluster correlation coefficient and our sample size calculation. To detect an increase in antenatal booking by 12 weeks gestation from 75% to 82%, with 90% power at the 5% significance level requires at least 751 individuals in each trial arm. To account for clustering (ICC = 0.005, mean cluster size 145, matching correlation = 0.1) requires nine clusters in the intervention group and in the control group, which equates to 1450 women per trial arm. Because this sample size is relatively small, and to guard against a substantial loss of power if a cluster is lost for any reason, we added one cluster to each group. As described, pairs of clusters were matched by the hospital used by those in the ward seeking maternity care and by baseline rate of antenatal booking by 12 weeks. This has been measured per ward and collected pre-randomisation.

### Process evaluation

Ongoing formative evaluation work will be conducted alongside Phases 1 and 2 of the intervention, in order to inform the development of the intervention structure, activities and materials. Research team members will conduct observations and interviews to document and analyse the activities in each intervention site during the stages of community engagement, co-design, intervention set-up and training of antenatal care champions. The UEL community engagement team will provide records on intervention set-up in each site, and co-host organisations will provide records on recruitment of antenatal care champions.

A process evaluation will be conducted for Phase 3 of the intervention, to explore its implementation. This integral process evaluation will run alongside the impact evaluation.

Process data will be used to examine intervention implementation, acceptability and reach, issues of context, as well as hypothesise possible causal pathways, in order to facilitate interpretation of outcome data. In line with recent Medical Research Council (MRC) guidance on process evaluations for complex interventions [26], this component of the trial will also enable refinement of the intervention logic model (see Fig. 1).

The process evaluation has four specific components. These are:

1. Documentation and analysis of the local social contexts in intervention and control sites

The mapping process described above in Phase 1 of the intervention will be expanded by the research team to document and analyse the local social context of the intervention and control sites and their main maternity providers, in order to identify events and influences that may hinder or support intervention implementation, or affect study outcomes. Information will also be recorded on all previous and current interventions/innovations regarding antenatal care that have been developed by maternity services, local authorities or community organisations for people living in the study sites

2. Documentation and analysis of intervention activities

Intervention activities will be documented and analysed for each site. Members of the research team will observe up to two purposively selected events in each of the intervention sites, where antenatal care champions engage with their local community about antenatal care. The UEL community engagement team and the co-host organisations will provide detailed records of intervention delivery and progress.

3. Interviews with antenatal care champions, co-host representatives and other stakeholders

The experiences of those taking part in the co-production and delivery of the intervention, and their perceptions regarding its impact on their communities, will be explored through qualitative research interviews. Up to 40 people, across three to four intervention ‘case study’ sites (final numbers dependent on data saturation), will be purposively sampled for individual interviews. Participants will be interviewed at the beginning and again towards the end of the intervention delivery phase. In the first interview, interviewees will be asked about their motivations for getting involved with the intervention, their expectations for how the intervention may be received within their local communities, their experiences and perspectives on the training process, and their early experiences with delivering the intervention messages around antenatal care. The second follow-up interview will be conducted with each participant approximately 6 months following their first interview, to address experiences with delivering the intervention and perceived intervention acceptability within the community. We will also aim to conduct interviews with those who cease their involvement with the intervention delivery while it is ongoing, in order to explore reasons for stopping. Interviews will also be conducted with local community midwives to gain insight into local maternity service provision and local barriers/enablers around access to antenatal care within the intervention site. Interviewees will receive a £10 voucher as reimbursement for their time. The qualitative research interviews will explore the social contexts within which the intervention is implemented, building a richer picture of intervention delivery and mechanisms of impact. The number of interviews to conduct in the remaining intervention sites, and who to interview, will be decided pragmatically and judiciously based on the findings from earlier sites.

4. Survey to assess exposure to the intervention and its influence

A sample of 400 women across the 20 trial sites will be surveyed to assess reach, exposure to and acceptability of the intervention. The survey will be conducted with women attending appointments at antenatal booking clinics 3 months following the start of the implementation of the intervention. We will aim to conduct this with an approximate ratio of 3:1 intervention to control women surveyed. Hospital staff will identify eligible women, when they attend the clinic, based on their postcode. Women who agree to participate, will be provided with the survey in written, self-administered form, with the offer of researcher or bilingual health advocate support where required. The survey will be translated into other languages that are common to women using that particular maternity service. The survey will not contain sensitive or personal questions regarding the woman’s pregnancy, but rather will focus on the woman’s first point of contact in her antenatal care pathway, whether she had heard of the intervention and whether it had any effect on her decision-making about the timing of antenatal care initiation. Participants will receive a £5 voucher as a ‘thank you’ for their participation.

Women who would like additional time to make a decision about participation in the survey will be provided with a link to an online version that they can complete at a future date. On completion they will be invited to send their address by email to the research team in order that a £5 voucher can be posted to them.

### Economic evaluation

A cost-consequences economic evaluation, will evaluate the effectiveness of the community-based intervention compared to current practice. Information collected during the process evaluation will be used to calculate the cost of the intervention for each ward including development and implementation costs. Health care resource use, collected for both trial arms, will include: antenatal bookings and appointments; antenatal admissions; mode of delivery with a focus on emergency cesareans; costs associated with pre-term births and low birth weight, maternal and infant deaths.

## Analysis

### Trial outcomes

A cluster-level analysis, appropriate to the analysis of matched cluster randomised trials, will be used with the maternity care providers for the NHS trusts participating in the study as fixed effects. We will use intention to treat principles. We will include individual level prognostic covariates if appropriate and ward-level estimates of baseline levels of outcomes as covariates. These will be chosen in advance of any analyses being conducted and documented in a full analysis plan. In additional analyses we will explore the use of instrumental variable techniques to incorporate some process measures as mediators of effect. Our primary analyses will consider primary and secondary outcomes data pertaining to births in the period 2–7 months post intervention start. Further secondary analyses will be conducted using data for births in the period 8–13 months post intervention completion to explore the maintenance of any effects and trends. A small number of sub group analyses may also be conducted using Stata software (version 14; StataCorp, College Station, TX, USA).

### Process evaluation

All qualitative interview data will be managed and coded using QSR International’s NVivo 11 qualitative data analysis software. Interview data will be subjected to thematic analysis. Codes will be applied to transcripts, to identify key themes and how these inter-relate in order to develop an analytical framework. Each transcript will also be coded to indicate the type of participant and electoral ward allowing analytical themes to be explored in relation to different groups’ experiences and to compare processes across intervention areas. Drawing on methods associated with ‘grounded theory’, constant comparisons will be made and deviant cases examined to refine the analysis.

Observation data will be recorded on a semi-structured proforma. Thematic analysis of this data will also be undertaken.

Data from the surveys will be analysed using the current version of IBM SPSS statistical software. Descriptive analysis will be conducted to assess the key themes relating to awareness and level of involvement with the intervention, attitudes towards it, and views on antenatal care.

### Economic evaluation

Costs and health care resource will be reported alongside primary and secondary outcomes for each trial arm. Missing data will be assumed to be missing at random and available case analysis used following the principles set out in the statistical analysis plan. We will report 95% confidence intervals calculated using bootstrapping.

A decision analytic model will also be developed to extrapolate the outcomes collected, antenatal admissions and emergency caesarean, their impact on costs and health outcomes and costs published in the literature for pre-term and low weight births. We will use a simplified decision analytical model to look at the benefits and disadvantages of the intervention, including the impact on the 12th completed week of pregnancy target, to assist NHS trusts with decisions about implementation; synthesising information from other sources where at all possible.

## Safety and trial conduct

There are no anticipated risks to study participants or to those involved with the intervention and the first outcomes data collection will occur after the intervention delivery is complete. Therefore, there will be no Data Monitoring Committee for this trial. This decision, made by the chief investigator (AH) and members of the Trial Steering Committee (TSC; see below), is due to the reliance on routine monitoring data that is provided at source in non-identifiable form. However, as in all interventions, there may be unanticipated risks and harms will be assessed through examination of outcomes at the two time points.

The UK MRC Guidelines on good clinical practice in trials [27] will be followed. The University of East London, the employer of AH, will act as the sponsor of this trial. The trial will be overseen by a TSC. This group will meet face to face once a year and will be responsible for overseeing the trial, ensuring scientific quality and clinical relevance, and adherence to ethics and research governance. All key collaborators on the trial will attend the TSC, as well as a range of experts who are not directly involved in the trial, including a chair with relevant expertise, a statistician and an economist. There will also be a maternity service user representative on the TSC. Bi-monthly trial management meetings, with the PCTU, will be held and study team meetings, involving AH, MW, MS, LS and CS, will take place once a month to oversee day-to-day progress.

## Dissemination

The findings of the trial will be presented at national and international conferences (e.g. Royal Colleges of Midwives annual conference, the International Confederation of Midwives Congress and relevant national public health conferences). They will also be published in peer-reviewed academic journals and in professional and practitioner journals. Findings will also be made available on the study website and in newsletters. Briefing papers to health care commissioners and managers and to service users via Maternity Voices Partnerships, will be prepared. We will use links with the Reproductive and Childbirth topic network to further disseminate throughout the NHS.

## Discussion

The Community REACH trial is one of a growing number of randomised controlled trials of public health interventions. The antenatal intervention being tested in the trial builds on evidence of effectiveness of lay or peer-delivered interventions when using community engagement strategies to provide health interventions to vulnerable or disadvantaged populations. A number of elements of the study will aid generalisability and scalability if effectiveness is shown. These elements include the integrated process and economic evaluations, the range of participating providers of maternity care in the trial, the flexibility of the intervention and the central involvement of local community members and community organisations. The application of a cluster randomised controlled trial design to the testing of an intervention that combines standardisation of overall approach with adaption to the local context will make a valuable contribution to the existing body of work on study design, as will the use of routine hospital data for outcomes analysis and the collaborative approach to research processes. If the intervention is shown to be effective it will be of benefit to those who received it and to society generally, in terms of improved health and associated reduction in cost to society and the NHS.

## Trial status

The study protocol reported here is version 2 (22 January 2016). There will be no active recruitment of participants to this trial as outcomes will be measured using anonymised routinely collected maternity services data. Individuals will be recruited to the integral process evaluation; recruitment began in June 2017 and is expected to finish in June 2018.

## Additional file

Additional file 1: SPIRIT Checklist: evaluation of community-level interventions to increase early initiation of antenatal care in pregnancy: Protocol for the Community REACH study, a cluster randomised controlled trial with integrated process and economic evaluations. (DOC 122 kb)

## Abbreviations

ANC: Antenatal Care
CLAHRC: Collaboration for Leadership in Applied Health Research and Care
IT: Information Technology
MRC: Medical Research Council
NIHR: National Institute for Health Research
PCTU: Pragmatic Clinical Trials Unit
PGfAR: Programme Grants for Applied Research
PI: Principal investigators
QMUL: Queen Mary’s University London
SPIRIT: Standard Protocol Items: Recommendations for Interventional Trials
TSC: Trial Steering Committee
UCL: University College London
UEL: University of East London

## Footnotes

1 An electoral ward in England is a subdivision of a local authority area, typically used for electoral purposes.
